# Supplementary material for: Genome-wide meta-analysis implicates mediators of hair follicle development and morphogenesis in risk for severe acne
Source: Nat Commun. 2018 Dec 12;9:5075. doi: 10.1038/s41467-018-07459-5 (PMC6290788; doi:10.1038/s41467-018-07459-5)
Supplement: Supplementary file 1 — Supplementary Information [file 41467_2018_7459_MOESM1_ESM.pdf]

# Supplementary Information

Genome-wide meta-analysis implicates mediators of hair follicle development and morphogenesis in risk for severe acne

Petridis *et al*.

## Supplementary Tables

**Supplementary Table 1**: Sex distribution of the study population.

| Status | Total | Males | Females |
|---|---|---|---|
| **cases** | 3,823 | 1,670 | 2,153 |
| **controls** | 16,144 | 7,232 | 8,912 |

**Supplementary Table 2**: Variants previously associated with severe acne.

| | | | | | | RAF | RAF | He *et al.* | | Navarini *et al.* | | | | |
|---|---|---|---|---|---|---|---|---|---|---|---|---|---|---|
| **Variant** | **Chr** | **Position (hg19)** | **Band** | **RA** | **PA** | **cases** | **controls** | **OR** | **95% CI** | **OR** | **95% CI** | $P_{meta}$ | **OR** | **95% CI** |
| rs747650 | 11 | 47,176,005 | 11p11.2 | T | C# | 0.32 | 0.32 | 1.24 | 1.16-1.34 | | | 0.601 | 1.01 | 0.97-1.06 |
| rs7531806 | 1 | 169,651,044 | 1q24.2 | G | A# | 0.44 | 0.43 | 1.22 | 1.12-1.28 | | | 0.206 | 1.03 | 0.98-1.07 |
| rs478304 | 11 | 65,494,260 | 11q13.1 | T* | G | 0.58 | 0.55 | | | 1.26 | 1.16-1.38 | $2.14 \times 10^{-9}$ | 1.14 | 1.09-1.19 |
| rs38055 | 5 | 52,560,644 | 5q11.2 | A* | G | 0.36 | 0.32 | | | 1.24 | 1.13-1.36 | $1.57 \times 10^{-14}$ | 1.19 | 1.14-1.24 |
| rs1159268 | 1 | 218,844,906 | 1q41 | A* | G | 0.38 | 0.35 | | | 1.15 | 1.05-0.26 | $4.74 \times 10^{-9}$ | 1.14 | 1.09-1.19 |

Chr, chromosome; RA, risk allele; PA, protective allele; RAF, risk allele frequency; OR, odds ratio; 95% CI, 95% confidence interval.
#previously reported acne risk allele [He *et al.*] [1]. * previously reported acne risk allele [Navarini *et al.*] [2].

**Supplementary Table 3**: Independent signals at four acne susceptibility regions.

| Variant | Chr | Position (hg19) | Band | RA | PA | RAF cases | RAF controls | Original analysis | | | Isolated analysis | | | Implicated gene |
|---|---|---|---|---|---|---|---|---|---|---|---|---|---|---|
| | | | | | | | | $P_{meta}$ | OR | 95% CI | $P_{meta}$ | OR | 95% CI | |
| rs1256580 | 1 | 219,199,380 | 1q41 | C | G | 0.18 | 0.15 | $1.23 \times 10^{-11}$ | 1.21 | 1.15-1.28 | $9.25 \times 10^{-10}$ | 1.19 | 1.13-1.26 | *TGFB2* |
| rs6684868 | 1 | 218,847,990 | | A | G | 0.49 | 0.46 | $2.42 \times 10^{-10}$ | 1.15 | 1.10-1.20 | $5.85 \times 10^{-9}$ | 1.14 | 1.09-1.19 | *TGFB2* |
| rs11118336 | 1 | 219,721,283 | | C | T | 0.45 | 0.42 | $1.49 \times 10^{-6}$ | 1.11 | 1.06-1.16 | $1.87 \times 10^{-7}$ | 1.12 | 1.07-1.17 | *TGFB2* |
| rs121908120 | 2 | 219,755,011 | 2q35 | A | T | 0.98 | 0.97 | $1.82 \times 10^{-12}$ | 1.94 | 1.61-2.33 | $2.60 \times 10^{-12}$ | 1.92 | 1.60-2.30 | *WNT10A* |
| rs72966077 | 2 | 219,759,229 | | C | T | 0.96 | 0.94 | $4.23 \times 10^{-7}$ | 1.33 | 1.19-1.48 | $2.30 \times 10^{-7}$ | 1.34 | 1.20-1.49 | *WNT10A* |
| rs144991069 | 11 | 64,827,708 | 11q13.1-13.2 | A | T | 0.02 | 0.01 | $5.00 \times 10^{-13}$ | 1.91 | 1.60-2.28 | $1.53 \times 10^{-11}$ | 1.84 | 1.54-2.19 | *OVOL1, MAP3K11* |
| rs61744384 | 11 | 65,387,378 | | T | A | 0.59 | 0.56 | $2.95 \times 10^{-11}$ | 1.16 | 1.11-1.21 | $6.10 \times 10^{-10}$ | 1.15 | 1.10-1.20 | *OVOL1, MAP3K11* |
| rs34560261 | 15 | 90,734,426 | 15q26.1 | C | T | 0.86 | 0.83 | $5.89 \times 10^{-15}$ | 1.32 | 1.23-1.41 | $3.30 \times 10^{-19}$ | 1.39 | 1.29-1.49 | *SEMA4B* |
| rs1533326 | 15 | 90,754,954 | | A | G | 0.67 | 0.66 | 0.00308 | 1.08 | 1.03-1.13 | $7.26 \times 10^{-8}$ | 1.15 | 1.10-1.22 | *SEMA4B* |

Chr, chromosome; RA, risk allele; PA, protective allele; RAF, risk allele frequency; OR, odds ratio; 95% CI, 95% confidence interval; Original analysis corresponds to unconditional associations; Isolated analysis corresponds to conditioning upon all remaining independent signals within the region of association

**Supplementary Table 4**: Bayesian fine-mapping of the 20 independent acne association signals.

| Band | Implicated gene | N within 95% CS | Variant with highest posterior ($V_{top}$) | BF *P* for $V_{top}$ |
|---|---|---|---|---|
| 1q41 | *TGFB2* | 8 | rs627486 | 0.20 |
| | *TGFB2* | 45 | rs1481361 | 0.06 |
| | *TGFB2* | 121 | rs11118336 | 0.10 |
| 2q35 | *WNT10A* | 5 | rs121908120 | 0.88 |
| | *WNT10A* | 56 | rs72966077 | 0.48 |
| 11q13.1-13.2 | *OVOL1, MAP3K11* | 5 | rs144991069 | 0.44 |
| | *OVOL1, MAP3K11* | 128 | rs61744384 | 0.47 |
| 15q26.1 | *SEMA4B* | 2 | rs34560261 | 0.66 |
| | *SEMA4B* | 145 | rs1533326 | 0.06 |
| 1q25.3 | *LAMC2* | 182 | rs10911268 | 0.11 |
| 1q32.1 | *LGR6* | 173 | rs788790 | 0.06 |
| 2p16.1 | *BCL11A* | 50 | rs2901000 | 0.07 |
| 2q14.2 | *GLI2* | 49 | rs1092479 | 0.08 |
| 4q27-28.1 | *FGF2* | 163 | rs4487353 | 0.03 |
| 5q11.2 | *FST* | 31 | rs629725 | 0.14 |
| 5q11.2 | | 29 | rs158639 | 0.11 |
| 7p14.1 | | 68 | rs7809981 | 0.48 |
| 8p23.1 | | 279 | rs28570522 | 0.15 |
| 11p15.3-15.2 | | 29 | rs2727365 | 0.17 |
| 22q11.23 | *SPECC1L* | 23 | rs28360612 | 0.46 |

Number of variants within the 95% credible set of causal variants; BF *P*, Bayes factor posterior probability of causality

**Supplementary Table 5**: Skin eQTLs from three datasets, having a colocalisation $P > 0.5$ with the acne association signals.

| Locus | Gene | Skin eQTL study | $P_{colocalisation}$ | Risk allele effect on gene expression |
|---|---|---|---|---|
| 11q13.1-13.2 | *MAP3K11* | MuTHER | 0.979 | ▼ |
| | *MAP3K11* | Not sun exposed GTEx | 0.967 | ▼ |
| | *MAP3K11* | Sun exposed GTEx | 0.956 | ▼ |
| | *KRT8P26* | Not sun exposed GTEx | 0.562 | ▲ |
| 15q26.1 | *SEMA4B* | Sun exposed GTEx | 0.999 | ▲ |
| | *SEMA4B* | Not sun exposed GTEx | 0.999 | ▲ |
| | *SEMA4B* | MuTHER | 0.983 | ▲ |
| 1q25.3 | *LAMC2* | MuTHER | 0.977 | ▲ |
| 1q32.1 | *PPP1R12B* | Sun exposed GTEx | 0.853 | ▼ |
| | *LGR6* | MuTHER | 0.749 | ▼ |
| 22q11.23 | *SPECC1L* | Sun exposed GTEx | 0.983 | ▲ |
| | *SPECC1L* | Not sun exposed GTEx | 0.509 | ▲ |
| | *POM121L9P* | Not sun exposed GTEx | 0.541 | ▲ |
| 4q27-28.1 | *FGF2* | Sun exposed GTEx | 0.809 | ▼ |

**Supplementary Table 6:** DEPICT output: 15 significant gene-sets along with genes within the genome wide significant loci that drive these associations. *P*-value relates to enrichment of gene-set membership among genes within identified acne susceptibility loci defined by $P_{meta} < 1 \times 10^{-5}$.

| **Gene set ID** | **Gene-set description** | ***P* value** | **Genes within genome wide significance regions** |
|---|---|---|---|
| MP:0000416 | sparse hair | $2.22 \times 10^{-6}$ | *OVOL1, LAMC2, BCL11A, WNT10A, LGR6, ATG2A, GLI2, PCNXL3, LTBP3* |
| KEGG | Small cell lung cancer | $7.02 \times 10^{-6}$ | *LAMC2, EHD1, WNT10A, CRTC3, TGFB2, CDC42BPG, MAP3K11* |
| ENSG00000172216 | CEBPB PPI subnetwork | $7.43 \times 10^{-6}$ | *RELA, CRTC3, BCL11A, MAP3K11, FST* |
| MP:0001675 | abnormal ectoderm development | $1.45 \times 10^{-5}$ | *FST, CDC42BPG, SPECC1L, TGFB2, BCL11A, LAMC2* |
| ENSG00000096696 | DSP PPI subnetwork | $1.70 \times 10^{-5}$ | *LAMC2, WNT10A, OVOL1, RELA, NAALADL1, CIB1, MAP3K11* |
| MP:0001065 | abnormal trigeminal nerve morphology | $3.80 \times 10^{-5}$ | *TGFB2, FST, FGF2, GLI2, WNT10A* |
| KEGG | Pathways in cancer | $3.92 \times 10^{-5}$ | *GLI2, FST, LAMC2, WNT10A, GABARAPL3, LGR6, SPECC1L, TGFB2* |
| MP:0002111 | abnormal tail morphology | $4.60 \times 10^{-5}$ | *FST, TGFB2, LGR6, WNT10A, LAMC2, GLI2* |
| ENSG00000136270 | TBRG4 PPI subnetwork | $5.09 \times 10^{-5}$ | *LAMC2, WNT10A, SLC22A5, SEMA4B* |
| GO:0033157 | regulation of intracellular protein transport | $5. 41 \times 10^{-5}$ | *SPECC1L, CDC42BPG, ADORA2A-AS1, LGR6, EHD1* |
| GO:0048634 | regulation of muscle organ development | $5.58 \times 10^{-5}$ | *LGR6, TGFB2, PPP1R3B, FST* |
| ENSG00000168487 | BMP1 PPI subnetwork | $7.63 \times 10^{-5}$ | *LAMC2, SEMA4B, TGFB2, WNT10A, C7orf10, GLI2* |
| GO:0046822 | regulation of nucleocytoplasmic transport | $8.38 \times 10^{-5}$ | *ADORA2A-AS1, CDC42BPG, SPECC1L, LGR6, EHD1* |
| MP:0004016 | decreased bone mass | $8.52 \times 10^{-5}$ | *SUGCT, GUCD1, GPHA2* |
| ENSG00000118495 | PLAGL1 PPI subnetwork | $1.06 \times 10^{-4}$ | *CRTC3* |

## Supplementary Figures

**Supplementary Figure 1**: Manhattan plot for acne meta-analysis.

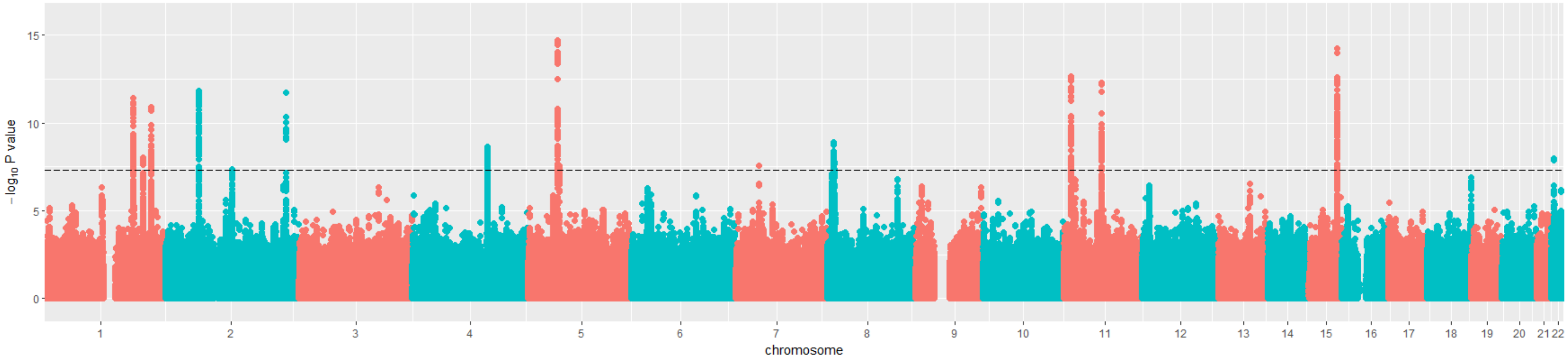

**Supplementary Figure 2**: Quantile-quantile (qq) plot for acne meta-analysis.

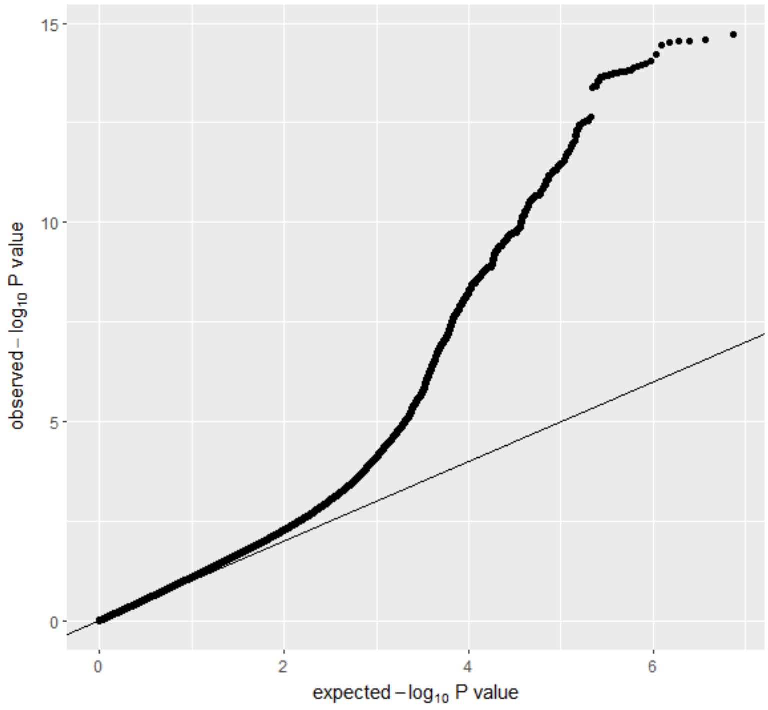

**Supplementary Figure 3**: Effect size (OR and 95% CI) of the 15 identified acne susceptibility loci separated by study.

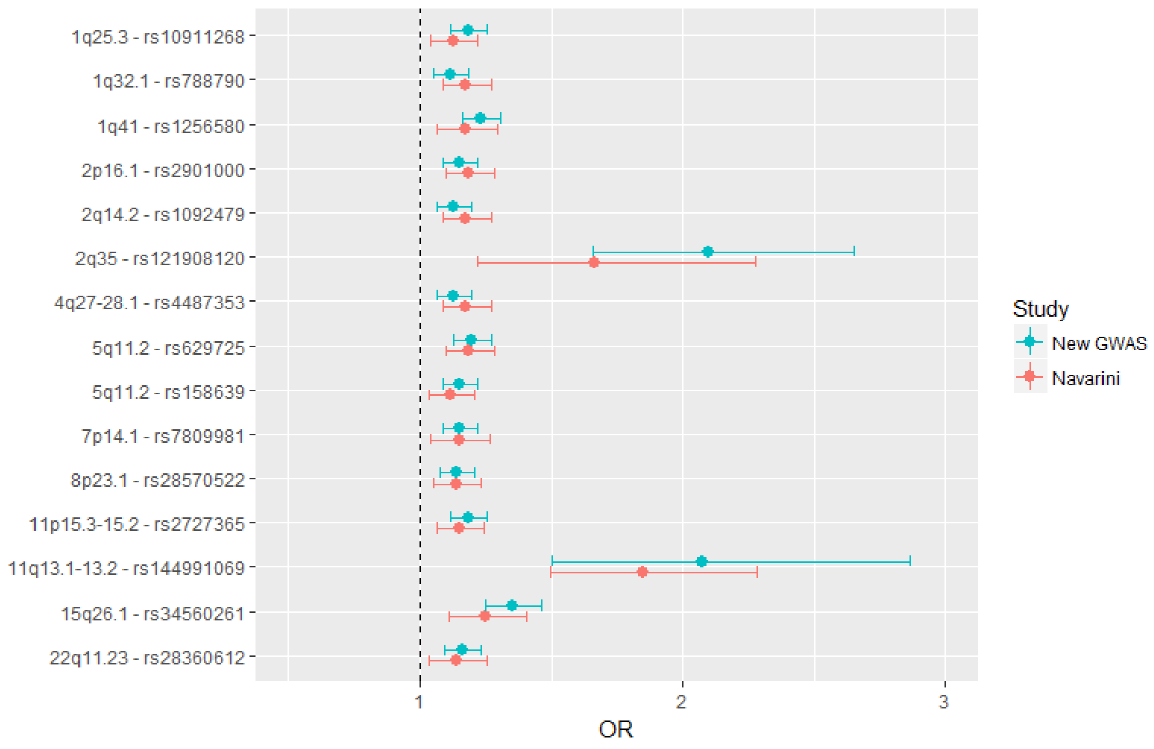

**Supplementary Figure 4**: Regional association plots for the region surrounding the 8p23.1 inversion; unconditional (top), and conditioned upon the inferred inversion genotypes (bottom).

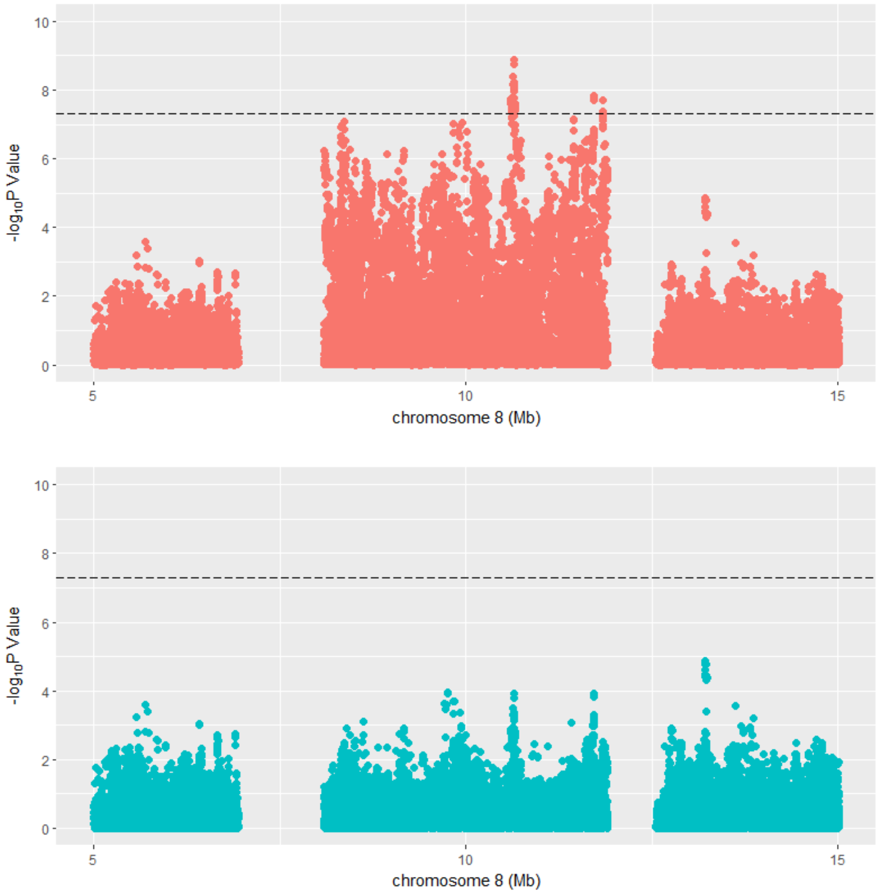

**Supplementary Figure 5**: Effect size (OR and 95% CI) of the 15 identified acne susceptibility loci separated per sex.

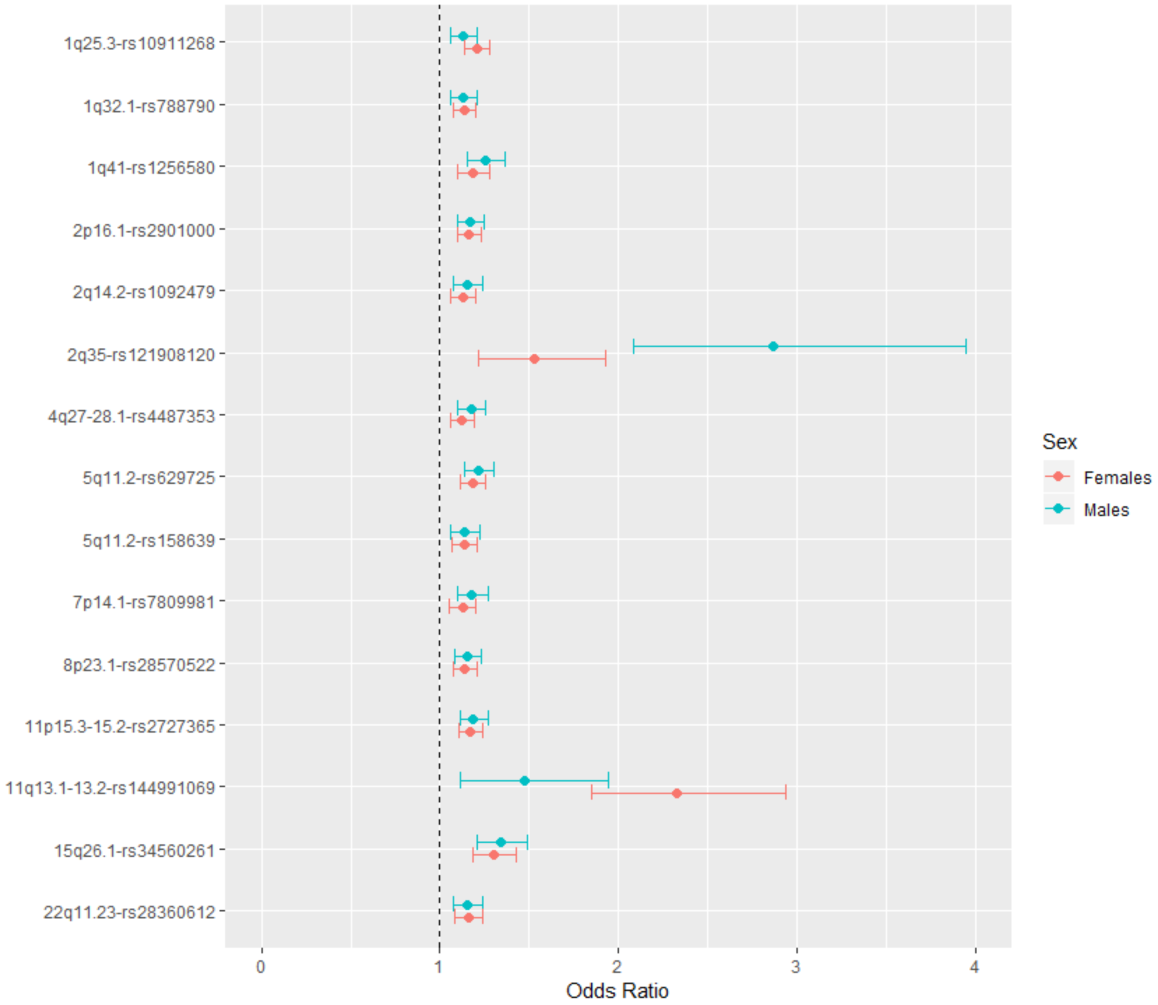

**Supplementary References**


1. He L*, et al.* Two new susceptibility loci 1q24.2 and 11p11.2 confer risk to severe acne. *Nature communications* **5**, 2870 (2014).

2. Navarini AA*, et al.* Genome-wide association study identifies three novel susceptibility loci for severe Acne vulgaris. *Nature communications* **5**, 4020 (2014).
