# Supplementary material for: Genome-wide meta-analysis implicates mediators of hair follicle development and morphogenesis in risk for severe acne
Source: Nat Commun. 2018 Dec 12;9:5075. doi: 10.1038/s41467-018-07459-5 (PMC6290788; doi:10.1038/s41467-018-07459-5)
Supplement: Supplementary file 3 — Description of Additional Supplementary Files [file 41467_2018_7459_MOESM3_ESM.pdf]

Description of Additional Supplementary Files

File Name: Supplementary Data 1
Description: Genetic correlation of severe acne with 175 other traits in the European population.
