# Supplementary material for: Genome-wide meta-analysis implicates mediators of hair follicle development and morphogenesis in risk for severe acne
Source: Nat Commun. 2018 Dec 12;9:5075. doi: 10.1038/s41467-018-07459-5 (PMC6290788; doi:10.1038/s41467-018-07459-5)
Supplement: Supplementary file 4 — Supplementary Data 1 [file 41467_2018_7459_MOESM4_ESM.docx]

**Supplementary Data 1**: Genetic correlation of severe acne with 175 other traits in the European population.

| **Trait** | **r_g_** | **SE** | ***P* value** |
| --- | --- | --- | --- |
| Crohns disease | -0.2219 | 0.0516 | 1.74 x 10^-5^ |
| Inflammatory Bowel Disease (Euro) | -0.2048 | 0.0501 | 4.42 x 10^-5^ |
| Bipolar disorder | -0.2282 | 0.0594 | 0.0001 |
| PGC cross-disorder analysis | -0.1852 | 0.0585 | 0.0016 |
| Triglycerides | 0.1874 | 0.0643 | 0.0036 |
| Waist-to-hip ratio | 0.1164 | 0.0418 | 0.0054 |
| ICV | 0.2339 | 0.097 | 0.0159 |
| Phospholipids in large HDL | -0.2016 | 0.0849 | 0.0175 |
| Concentration of large HDL particles | -0.1966 | 0.0846 | 0.0201 |
| Forced expiratory volume in 1 second (FEV1)/Forced Vital capacity(FVC) | 0.1903 | 0.0819 | 0.0201 |
| Isoleucine | 0.2576 | 0.1138 | 0.0235 |
| Free cholesterol in large HDL | -0.2111 | 0.0941 | 0.0248 |
| Total lipids in large HDL | -0.1876 | 0.0856 | 0.0284 |
| Forced expiratory volume in 1 second (FEV1) | 0.1129 | 0.0559 | 0.0435 |
| Total cholesterol in large HDL | -0.1924 | 0.0972 | 0.0477 |
| Free cholesterol in medium HDL | -0.2275 | 0.1164 | 0.0507 |
| HDL cholesterol | -0.0981 | 0.0509 | 0.0537 |
| Height_2010 | -0.0832 | 0.0434 | 0.0553 |
| Total cholesterol in HDL | -0.2056 | 0.1075 | 0.0558 |
| Anorexia Nervosa | -0.1005 | 0.0531 | 0.0586 |
| Ulcerative colitis | -0.1272 | 0.0674 | 0.0593 |
| Primary biliary cirrhosis | -0.1655 | 0.0891 | 0.0631 |
| Cholesterol esters in large HDL | -0.1632 | 0.0904 | 0.0709 |
| Mean diameter for HDL particles | -0.1693 | 0.0945 | 0.0731 |
| Forced expiratory volume in 1 second (FEV1)/Forced Vital capacity(FVC) | 0.1065 | 0.0598 | 0.0749 |
| Concentration of large VLDL particles | 0.1496 | 0.0844 | 0.0761 |
| Systemic lupus erythematosus | -0.2148 | 0.1225 | 0.0795 |
| Phospholipids in very large HDL | -0.1949 | 0.112 | 0.082 |
| HbA1C | -0.1402 | 0.0821 | 0.0876 |
| Forced expiratory volume in 1 second (FEV1) | 0.1216 | 0.0714 | 0.0883 |
| Rheumatoid Arthritis | -0.0894 | 0.0534 | 0.0939 |
| Childhood IQ | 0.1371 | 0.0821 | 0.0948 |
| Obesity class 2 | 0.0898 | 0.056 | 0.1091 |
| Phospholipids in medium HDL | -0.1667 | 0.1061 | 0.1163 |
| Mean Putamen | 0.1024 | 0.0655 | 0.1182 |
| LDL cholesterol | -0.0929 | 0.0596 | 0.1193 |
| Ferritin | 0.1988 | 0.1291 | 0.1235 |
| Squamous cell lung cancer | 0.1802 | 0.1185 | 0.1282 |
| Forced Vital capacity(FVC) | 0.0737 | 0.0506 | 0.1451 |
| Age of first birth | -0.077 | 0.0537 | 0.1516 |
| Triglycerides in chylomicrons and largest VLDL particles | 0.1384 | 0.0973 | 0.1549 |
| Triglycerides in very small VLDL | 0.127 | 0.0912 | 0.1639 |
| Total lipids in chylomicrons and largest VLDL particles | 0.1347 | 0.097 | 0.1648 |
| Creatinine | 0.1119 | 0.0821 | 0.1731 |
| Neo-openness to experience | 0.1449 | 0.1064 | 0.1733 |
| Obesity class 1 | 0.07 | 0.0525 | 0.1823 |
| Triglycerides in small VLDL | 0.1212 | 0.0918 | 0.187 |
| Depressive symptoms | -0.1011 | 0.0777 | 0.1929 |
| Phospholipids in chylomicrons and largest VLDL particles | 0.1301 | 0.1008 | 0.1966 |
| Mean Pallidum | -0.1299 | 0.1015 | 0.2007 |
| Serum total triglycerides | 0.1099 | 0.0859 | 0.201 |
| Phospholipids in very large VLDL | 0.1132 | 0.0891 | 0.2036 |
| Extreme waist-to-hip ratio | 0.1116 | 0.0879 | 0.2045 |
| Free cholesterol in large VLDL | 0.0988 | 0.0796 | 0.2146 |
| Citrate | 0.1253 | 0.1027 | 0.2221 |
| Cholesterol esters in large VLDL | 0.0918 | 0.0757 | 0.2256 |
| Extreme height | -0.0696 | 0.0576 | 0.227 |
| Triglycerides in large VLDL | 0.1015 | 0.0856 | 0.2357 |
| Lung cancer (all) | 0.102 | 0.0868 | 0.2396 |
| Waist circumference | 0.0501 | 0.0431 | 0.2451 |
| Triglycerides in very large VLDL | 0.0924 | 0.0798 | 0.2465 |
| Asthma | -0.1216 | 0.1059 | 0.2506 |
| Total lipids in very small VLDL | 0.1199 | 0.1044 | 0.2507 |
| Birth weight | -0.0516 | 0.0456 | 0.2574 |
| Total lipids in very large VLDL | 0.0927 | 0.0822 | 0.2592 |
| Concentration of medium VLDL particles | 0.0876 | 0.078 | 0.2614 |
| Urinary albumin-to-creatinine ratio (non-diabetes) | -0.0933 | 0.0859 | 0.2772 |
| Infant head circumference | -0.0938 | 0.0866 | 0.279 |
| Urinary albumin-to-creatinine ratio | -0.097 | 0.0899 | 0.2809 |
| Total cholesterol in large VLDL | 0.0883 | 0.0822 | 0.2829 |
| Total lipids in large VLDL | 0.0893 | 0.0839 | 0.2874 |
| Forced expiratory volume in 1 second (FEV1)/Forced Vital capacity(FVC) | 0.0657 | 0.0621 | 0.2896 |
| Fasting glucose main effect | -0.0779 | 0.0737 | 0.2902 |
| Concentration of small VLDL particles | 0.0852 | 0.0808 | 0.2914 |
| Platelet count | 0.064 | 0.0608 | 0.2926 |
| Concentration of very small VLDL particles | 0.1034 | 0.0988 | 0.2951 |
| Phospholipids in medium VLDL | 0.0904 | 0.0866 | 0.2964 |
| Mean diameter for VLDL particles | 0.0894 | 0.0864 | 0.301 |
| Triglycerides in IDL | 0.1077 | 0.1066 | 0.3125 |
| Childhood obesity | 0.0641 | 0.0635 | 0.3127 |
| Triglycerides in medium VLDL | 0.0896 | 0.09 | 0.3194 |
| Lung cancer | 0.0755 | 0.0765 | 0.3238 |
| Alzheimers disease | -0.101 | 0.104 | 0.3315 |
| Major depressive disorder | -0.0824 | 0.0852 | 0.3336 |
| Phospholipids in large VLDL | 0.0842 | 0.0883 | 0.3406 |
| Total lipids in small VLDL | 0.0772 | 0.0819 | 0.3462 |
| Fathers age at death | 0.0988 | 0.1062 | 0.352 |
| Forced expiratory volume in 1 second (FEV1) | 0.0798 | 0.0864 | 0.3555 |
| Glucose | 0.0831 | 0.0902 | 0.3565 |
| Apolipoprotein B | 0.1032 | 0.1128 | 0.3599 |
| Phospholipids in small VLDL | 0.0838 | 0.0925 | 0.3651 |
| Free cholesterol in medium VLDL | 0.0791 | 0.0881 | 0.3691 |
| Child birth length | 0.077 | 0.0877 | 0.3802 |
| Free cholesterol in small VLDL | 0.0815 | 0.0931 | 0.3811 |
| Omega-3 fatty acids | 0.0939 | 0.1082 | 0.3851 |
| Extreme bmi | 0.06 | 0.0697 | 0.3893 |
| Total lipids in medium VLDL | 0.0657 | 0.0772 | 0.3948 |
| Age at Menopause | 0.0444 | 0.0526 | 0.3991 |
| Total cholesterol in medium VLDL | 0.0715 | 0.0848 | 0.3995 |
| Valine | 0.0939 | 0.1135 | 0.4077 |
| Difference in height between childhood and adulthood; age 8 | -0.0866 | 0.1061 | 0.414 |
| Concentration of very large VLDL particles | 0.0726 | 0.0918 | 0.4293 |
| Chronotype | -0.0368 | 0.0522 | 0.4806 |
| Alanine | 0.0646 | 0.0947 | 0.4951 |
| Mean Thalamus | -0.0858 | 0.1259 | 0.4957 |
| Cholesterol esters in medium VLDL | 0.0529 | 0.078 | 0.4977 |
| Age at Menarche | -0.0268 | 0.0402 | 0.5044 |
| Cigarettes smoked per day | -0.0607 | 0.0918 | 0.5082 |
| Glycoprotein acetyls; mainly a1-acid glycoprotein | 0.0601 | 0.0951 | 0.527 |
| Mothers age at death | 0.0599 | 0.0952 | 0.529 |
| Sitting height ratio | -0.0534 | 0.0857 | 0.5329 |
| Body mass index | 0.0285 | 0.0474 | 0.547 |
| HOMA-IR | 0.0736 | 0.1235 | 0.5512 |
| College completion | 0.0335 | 0.0562 | 0.5517 |
| Age of smoking initiation | -0.0785 | 0.1508 | 0.6028 |
| Concentration of small LDL particles | 0.0589 | 0.1144 | 0.6068 |
| Leptin_adjBMI | -0.0516 | 0.1012 | 0.61 |
| HOMA-B | 0.0589 | 0.1159 | 0.6112 |
| Parkinsons disease | -0.0348 | 0.0685 | 0.6115 |
| Phospholipids in very small VLDL | 0.059 | 0.1176 | 0.6156 |
| Phospholipids in IDL | 0.0662 | 0.1341 | 0.6215 |
| 18:2 linoleic acid (LA) | -0.0554 | 0.1145 | 0.6287 |
| Total Cholesterol | -0.0258 | 0.0537 | 0.6302 |
| Mean platelet volume | -0.0355 | 0.0738 | 0.6303 |
| Concentration of IDL particles | 0.0614 | 0.128 | 0.6317 |
| Total cholesterol in small VLDL | 0.0465 | 0.1001 | 0.6426 |
| Overweight | 0.0243 | 0.0536 | 0.6505 |
| Total cholesterol in small LDL | 0.0573 | 0.1265 | 0.6506 |
| Total lipids in small LDL | 0.0523 | 0.118 | 0.6577 |
| Mean Caudate | -0.0349 | 0.0804 | 0.664 |
| Phospholipids in large LDL | 0.0569 | 0.1334 | 0.6695 |
| Apolipoprotein A-I | -0.054 | 0.1271 | 0.671 |
| Concentration of chylomicrons and largest VLDL particles | 0.0412 | 0.101 | 0.6833 |
| Child birth weight | -0.0342 | 0.0847 | 0.6869 |
| Subjective well being | 0.0311 | 0.0789 | 0.6935 |
| Total lipids in IDL | 0.0492 | 0.1298 | 0.7047 |
| Phospholipids in medium LDL | 0.0428 | 0.1212 | 0.724 |
| Leptin_not_adjBMI | 0.0288 | 0.0868 | 0.7399 |
| Sleep duration | -0.0266 | 0.0814 | 0.7435 |
| Years of schooling (proxy cognitive performance) | -0.0166 | 0.0511 | 0.7454 |
| 22:6 docosahexaenoic acid | 0.0368 | 0.114 | 0.7468 |
| Concentration of medium LDL particles | 0.0397 | 0.1244 | 0.7497 |
| Former vs Current smoker | -0.0335 | 0.1098 | 0.76 |
| Fasting insulin main effect | 0.0253 | 0.0836 | 0.7621 |
| Obesity class 3 | -0.0189 | 0.0708 | 0.789 |
| Description of average fatty acid chain length; not actual carbon number | -0.029 | 0.1133 | 0.7982 |
| Free cholesterol in large LDL | 0.0338 | 0.1334 | 0.8002 |
| Concentration of large LDL particles | 0.0318 | 0.1291 | 0.8057 |
| Total cholesterol in large LDL | 0.0319 | 0.1299 | 0.8059 |
| Total lipids in medium LDL | 0.0301 | 0.123 | 0.8066 |
| Total cholesterol in LDL | 0.0307 | 0.1277 | 0.81 |
| Height; Females at age 10 and males at age 12 | -0.0174 | 0.0744 | 0.8148 |
| Average number of double bonds in a fatty acid chain | 0.0206 | 0.0946 | 0.8274 |
| Years of schooling 2013 | -0.0107 | 0.0501 | 0.831 |
| Total lipids in large LDL | 0.0229 | 0.1296 | 0.8598 |
| Neuroticism | 0.0159 | 0.0947 | 0.8665 |
| Glutamine | -0.0186 | 0.1154 | 0.872 |
| Celiac disease | -0.0138 | 0.0866 | 0.8731 |
| Mean Hippocampus | 0.0184 | 0.1294 | 0.8867 |
| Total cholesterol in IDL | 0.0174 | 0.1337 | 0.8962 |
| Neuroticism | 0.0132 | 0.1037 | 0.8984 |
| Number of children ever born | 0.0058 | 0.0475 | 0.9028 |
| Cholesterol esters in medium LDL | 0.0134 | 0.1231 | 0.9131 |
| Ever vs never smoked | 0.0066 | 0.0654 | 0.92 |
| Type 2 Diabetes | -0.0074 | 0.0802 | 0.9261 |
| Years of schooling 2016 | -0.0032 | 0.0347 | 0.9268 |
| Parents age at death | 0.0117 | 0.1354 | 0.9313 |
| Free cholesterol in IDL | 0.01 | 0.1228 | 0.9353 |
| Forced Vital capacity(FVC) | 0.0053 | 0.0691 | 0.9387 |
| Urate | -0.0032 | 0.044 | 0.9422 |
| Difference in height between adolescence and adulthood; age 14 | -0.0095 | 0.1317 | 0.9427 |
| Total cholesterol in medium LDL | 0.0089 | 0.1263 | 0.9438 |
| Autism spectrum disorder | 0.0037 | 0.1023 | 0.971 |
| Cholesterol esters in large LDL | 0.0043 | 0.1283 | 0.9734 |
| Hip circumference | 0.0012 | 0.0427 | 0.9776 |

r_g_, SNP genetic correlation between severe acne and reported trait; SE, Standard error;
